# UTMD promoted local delivery of miR-34a-mimic for ovarian cancer therapy

**DOI:** 10.1080/10717544.2021.1955041

**Published:** 2021-07-28

**Authors:** Yue Li, Meng Du, Jinghui Fang, Jia Zhou, Zhiyi Chen

**Affiliations:** a The First Affiliated Hospital, Medical Imaging Centre, Hengyang Medical School, University of South China, Hengyang, Hunan, China; b Institute of Medical Imaging, University of South China, Hengyang, China; c Laboratory of Ultrasound Molecular Imaging, The Third Affiliated Hospital of Guangzhou Medical University, Guangzhou, China; d The First Affiliated Hospital, Department of Ultrasound Medicine, Hengyang Medical School, University of South China, Hengyang, Hunan, China

**Keywords:** Ultrasound, microbubbles, multi-parameters, MiR-34a mimic, cancer therapy

## Abstract

MicroRNA-mediated gene therapy is emerging as a promising method for the treatment of ovarian cancer, but the development of miRNA mimic delivery vectors is still in its infancy, where the safety and efficacy of miR-34a-mimic remain unknown. Ultrasound-targeted microbubble destruction (UTMD) can be an effective and minimally invasive tool for the delivery of miR-34a-mimic *in vitro* and *in vivo*. Here, we describe a high-efficiency gene delivery strategy by using miR-34a-mimic loaded folate modified microbubbles (miR-34a-FM) with a portable ultrasonic irradiation system. Ultrasonic parameters, including acoustic intensity (AI), exposure time (ET) and duty cycle (DC), were optimized and the optimal acoustic condition (1.0 W/cm^2^, 20 s, and 15% DC) was used to deliver miRNA-34a into cells *in vitro.* MiR-34a mimic was successfully introduced into the cytoplasm and was found to inhibit proliferation and induce apoptosis of SK-OV-3 cells. Next, miR-34a-mimic was delivered to tumor tissue via UTMD, inhibiting tumor growth and prolonging the survival time of mice. In summary, UTMD-mediated miR-34a-mimic delivery has potential application in the clinical treatment of ovarian cancer.

## Introduction

MicroRNAs (miRNA) are small, non-coding, single-stranded RNAs and are usually 17–23 nucleotides (nt) long, which can modulate the expression of multiple genes post-transcriptionally in animals (Bartel, 2009). Multiple miRNAs contribute to maintaining a proper balance of physiological and pathological processes, including proliferation, cell cycle, and metabolism (Krol et al., 2010). Accordingly, miRNAs have shown great potential to serve as new cancer therapy targets, which was associated with cancer cell proliferation, invasion, migration, drug resistance, and apoptosis (Ventura & Jacks, 2009). miR-34a is a naturally occurring tumor suppressor that is absent or expressed at reduced levels in a variety of tumor types (He et al., 2007). miR-34a activity can also be enhanced using miR-34a-mimic. Ideally, miRNA mimics should be injected into the bloodstream and absorbed only by specific tissues or cells. However, synthetic naked miRNA mimic was unstable in nuclease-rich serum, and there remain unknown risks from injection into the bloodstream (Su et al., 2018; Xiao et al., 2018; Zeng et al., 2019). So far, the development of miR-34a-mimic delivery vectors is still in its infancy and localized delivery of miR-34a-mimic remains a challenge.

Lipid-based delivery systems have been used to introduce miR-34 into tumor tissue. The use of nanocarriers, for example, liposomes, is a promising solution to the problem (Jang et al., 2016). The liposomal formulation allows for delivery of the nucleic acid to the tumor site with natural biological compatibility and good stability. Unfortunately, there are some impediments to the clinical application of liposomes, such as low specificity and short circulating time of the blood system. Only one clinical trial so far evaluating the safety, pharmacokinetics, and pharmacodynamics of a liposomal nanoparticle formulation of a naturally occurring tumor suppressor miR34a (MRX34) (ClinicalTrials.gov Identifier: NCT01829971), a synthetic, double-stranded miR-34a mimic that encapsulated in a liposomal nanoparticle (Hong et al., 2020). To deliver them, a robust and versatile delivery system is needed to protect miR-34a mimic encapsulated in the delivery carrier while circulating in the body and deliver to the cytoplasm from the endo-lysosomes after internalization into the cancer cells.

Recently, we presented gas-filled microbubbles (MBs) as a drug delivery vehicle to enhance gene localized delivery with Ultrasound (US) (Liufu et al., 2019, 2020; Zhang et al., 2020). MBs are regarded as contrast agents and gene vectors simultaneously to play the main role in ultrasound-targeted microbubble destruction (UTMD) (Meijering et al., 2009; Meng et al., 2019). US stimulation mediates MBs oscillated and induced rapid disruption of the cell membrane to form transient pores (Sonoporation), leading to cell endocytosis or promotes drug transport through the pores into the cytoplasm (Qiu et al., 2012; Helfield et al., 2016; Rong et al., 2018). Most of the MBs, ranging in diameter from 1 to 10 μm, consist of inert gas as a core and a variety of shells (phospholipids, proteins, surfactants, or biocompatible polymers). These gas-filled spheres can be given a positive net charge and loaded with molecules such as siRNA and miRNA. Encapsulating genes in MBs can prevent them from degradation by nucleases, which was expected to be an ideal delivery vector. An additional advantage of this MBs-based US-mediated gene therapy strategy is the possibility of localized release in deep tissues and delivery of the loaded compound via real-time US imaging. Folate (FA) receptor is a single-chain glycoprotein anchored on the surface of the cell membrane, which was overexpressed in tumor tissue. The expression level of the FA receptor increases with the progression of the tumor, so it can be used as a tumor target.

Herein, in this study, we synthesized miR-34a-mimic-loaded MBs as the miRNA vectors and performed modification with FA. After US-mediated MBs disruption in the tumor site, the miR-34a-mimic could reach the tumor tissue with the aid of cavitation and gene delivery through FA-mediated cellular endocytosis. In our previous study, we determined that the exogenous introduction of miR-34a-mimic-loaded MBs *in vitro* resulted in the apoptosis of breast cancer cells and reduced cell proliferation (Zhang et al., 2020). However, the efficacy of miR-34a-mimic in the treatment of ovarian cancer remains unknown. We hypothesized the Notch1 gene as a therapeutic target by using miR-34a to knock down its expression, thereby inducing Bcl-2/Caspase-3 signaling pathway mediated apoptosis and inhibiting the growth of ovarian cancer. The aim of this study was to establish a strategy for local miR-34a-mimic delivery using UTMD and to evaluate the efficacy of small RNA-mediated ovarian cancer therapy.

## Materials and methods

### Preparation of lipid-coated miR-34a-mimic-FA-MBs

Lipid-coated ultrasound contrast agents were prepared following the protocol described previously on preparing cationic MBs with some modifications (Yang et al., 2018). In brief, 1,2-distearoyl-sn-glycero-3-phosphoethanolamine-N-[folate(polyethylene glycol)] (DSPE-PEG2000-FA) (Xi’an Ruixi Biological Technology Co., Ltd.l) was utilized to create FA receptor-targeted MBs (FM) by mixing dipalmitoyl-phosphatidyl-choline (DPPC) (Avanti Polar Lipids, AL, USA) and 1,2-dipalmitoyl-3-trimethylammonium-propane (DOTAP) (Avanti Polar Lipids, AL, USA) in chloroform at a molar ratio of 2:9:1. After drying over 2 h in a 65 °C water bath, lipid films were generated at the bottom of glass vials. Then, the lipid films were hydrated at 65 °C with 800 mL 1% (w/w) glycerol-Dulbecco’s phosphate-buffered saline (DPBS, Gibco, USA) mixture. Subsequently, miR-34a-mimic (0 ∼ 30 μg) (Genepharma, Shanghai, China) was added to the lipid film complexes, and the mixture was degassed and refilled with perfluoropropane (C_3_F_8_). The miR-34a-mimic-FA-MBs (miR-34a-FM) were formed by intense mechanical shaking using an agitator for 45 s and were then centrifuged at 2000 *g* for 1 min to separate unattached mimic.

### Characterization of miR-34a-mimic-FA-MBs

The zeta potential and size distribution of the miR-34a-FM were measured by a Zetasizer (Malvin, FL, USA). To visualize the miR-34a-mimic loaded onto the shell of MBs, miR-34a-mimic was labeled with Cy5 red fluorescence dye (GenePharma, Shanghai, China) and was used to prepare Cy5-miR-34a-FM. The acoustic stability of the miR-34a-FM was evaluated by measuring the echogenicity from contrast-enhanced ultrasound (CEUS) and B-mode images. The miR-34a-FM were systematically delivered through the tail vein of tumor-bearing mice and imaged with a 5 ∼ 12 MHz sonographic system (IU22, Philips, Netherlands).

### Cell culture

Human ovarian cancer cells SK-OV-3 were purchased from the American Type Culture Collection (ATCC, USA) and cultured in accordance with the ATCC guidelines before use. SK-OV-3 cells were maintained in McCoy’s 5a Medium Modified (Gibco, USA) supplemented with 10% fetal bovine serum (FBS, Gibco, USA) and 1% penicillin-streptomycin (Gibco, USA) at 37 °C in 5% CO_2_ atmosphere.

### US parameters

Studies had shown that fluorescent molecules (e.g. FITC) can penetrate to the cytosol through the pores caused by UTMD (De Cock et al., 2015; Song et al., 2015; Bhutto et al., 2018). To examine the optimum parameters for UTMD-enhanced miR-34a-mimic delivery, 150 kDa Fluorescein isothiocyanate-labeled dextran (FITC-dextran, Sigma-Aldrich Co. LLC, Shanghai, China) was used as model molecules for the evaluation of UTMD-mediated sonoporation efficiency. A total of 1 × 10^5^ cells were seeded in a 24 well plate in 500 µL medium (McCoy’s 5a medium with 10% FBS). After 24 h, when the cell confluency reached approximately 80%, the medium was exchanged with 500 μL of serum-free Opti-MEM medium (Gibco, USA). For the experiment, 1 × 10^8^ miR-34a-FM was added to the medium. The cell dish was sonicated by a US system (SXUltrasonic, Shenzhen, China) with a 1 MHz unfocused ultrasonic probe under different parameters including acoustic intensity (AI, 0.6 ∼ 1.2 W/cm^2^), duty cycle (DC, 5 ∼ 20%), and exposure time (ET, 20 ∼ 80 s) to facilitate the transfection of UTMD. 15 min after US exposure, cells were washed with DPBS and the percentage of FITC-dextran to cells was measured by flow cytometer (Attune^®^ NxT Flow Cytometer, Invitrogen, USA). 24 h after UTMD treatment of SK-OV-3 cells, a cell counting kit-8 (CCK-8, BestBio, China) assay was performed to evaluate cell viability. A final concentration of 10% CCK-8 reagent was added to the cells and the cells were incubated in a 5% CO_2_, 37 °C incubators for 2 h. The absorbance at 450 nm was measured by an ELx808 Absorbance Microplate Reader (Bio-Tek, USA).

### Scanning electron microscope

The alterations in cell membrane morphology attributed to sonoporation were observed by scanning electron microscopy (SEM). At different times after US treatment (1 min, 30 min, and 60 min), cells were washed twice with DPBS and fixed with 4% glutaraldehyde. SEM (S-3000N, Hitachi Ltd., Japan) was employed to observe the morphological changes of cells.

### Intracellular delivery of miR-34a-FM

The Intracellular delivery of miR-34a-FM via UTMD was investigated using confocal microscopy and flow cytometry at different time points. SK-OV-3 cells (1 × 10^5^ cells/well) were inoculated onto a 24 well plate with glass coverslips at the bottom. The next day, microbubbles that contained Cy5-miR-34a-FM (300 nM) were added to well plates and treated with UTMD. 12 h later, the glass coverslips were washed twice with DPBS and fixed with 4% paraformaldehyde. After washing twice with DPBS, the cells were stained with DAPI solution. The intracellular localization of the Cy5-miR-34a mimic was analyzed by a Pannoramic MIDI scanner (3DHISTECH, Budapest, Hungary).

### Apoptosis assay

Apoptosis was evaluated by an Annexin V-FITC/propidium iodide (PI) cell apoptosis detection kit (BestBio, China) as previously described. The transfected ovarian cancer SK-OV-3 cells (5 × 10^5^) were seeded in 6 well plates and subjected to incubation for 12 h. After US treatment, SK-OV-3 cells were collected and stained with PI and Annexin V for 15 min away from light and analyzed by flow cytometry.

### The qRT-PCR analysis

Total RNA was purified, and reverse transcribed by a miR-34a-specific primer (GTCGTATCCAGTGCAGGGTCCGAGGTATTCGCACTGGATACGACAACAAC) and a U6-specific primer (AACGCTTCACGAATTTGCGT). U6 was used as a reference gene. qPCR was performed to quantify miR-34a in SK-OV-3 cells according to the manufacturer’s instructions. The primer pair for miR-34a was as follows: miR-34a-forward, GGGTGGCAGTGTCTTAGC; miR-34a-reverse, GTGCAGGTCCGAGGT. The primer pair for U6 was as follows: U6-forward, CTCGCTTCGGCAGCACA; U6-reverse, AACGCTTCACGAATTTGCGT. The relative expression of miR-34a was calculated according to the 2^−ΔΔCT^ method.

### Cell invasion assays

In serum-free McCoy’s 5a medium, 5 × 10^5^ cells/mL SK-OV-3 cells were suspended, and 100 μL (5 × 10^4^ cells) was added to the upper chamber of the Transwell chamber (Corning Incorporated, Corning, NY, USA), and 700 μL of complete medium was added to the lower chamber. After incubation overnight at 37 °C, 5% CO_2_, cells were fixed with 4% paraformaldehyde for 15 min and washed with DPBS. After staining with crystal violet for 10 min, cells were washed with DPBS three times, and photos were taken under the microscope.

### Western blot analysis

Cells were lysed and total proteins were extracted using RIPA buffer. The sample was separated by sodium dodecyl sulfate-polyacrylamide gel electrophoresis (SDS-PAGE) and transferred to a polyvinylidene fluoride (PVDF) membrane. The PVDF membranes were blocked with 5% Bovine Serum Albumin (BSA, Sigma, Shanghai, China) for 1 h at room temperature and were then incubated with primary antibodies (Notch1, Bcl-2, and Caspase-3) overnight. 1 × Tris Buffered Saline Tween (TBST) was washed three times and a rabbit IgG-HRP antibody was added as the secondary antibody. The protein expression levels of Notch1, Bcl-2, and Caspase-3 protein were normalized to the GAPDH level.

### Tumor model

Female BALB/c nude mice (4–5 weeks, 18–20 g, Medical Experimental Animal Center, Guangzhou, China) were used in this study. All animal studies were performed under the protocol approved by the Care and Use of Laboratory Animals. SK-OV-3 tumor cells were resuspended in DPBS and were then subcutaneously injected at the right flank of the mice (3 × 10^6^ cells in 100 µL DPBS). Ten days after the injection, the volume of the xenograft tumor was calculated every two days.

### *In vivo* US molecular imaging

Mice were anesthetized with 1% pentobarbital and the body temperature was kept constant at 37 °C in a living imaging system (IVIS Lumina XRMS Series III, PerkinElmer, USA). 100 µL Cy5-miR-34a-FM (1 × 10^9^/mL) that contained 30 µg Cy5-miR-34a-mimic was intravenously administered through the tail vein (body weight: 20 ∼ 22 g; injection time: 3 s). Every mouse was treated with US (2.0 W/cm^2^, 50% Duty cycle, 1 min) and was subsequently imaged after treatment. Living Image 4.5 software was utilized (PerkinElmer, USA). Subsequently, the mice were sacrificed, the subcutaneous tumors and other organs were separated to observe the distribution of red fluorescence.

### *In vivo* antitumor evaluation

After the tumor size had reached 100 mm^3^, mice were randomly divided into four groups (four mice per group). The mice received administration of miR-34a, miR-34a-FM, or saline through the tail vein, and US-mediated gene delivery was analyzed as described above. The size of the tumor was recorded every 3 days (the calculation formula as follows). The tumor growth curves were delineated.

### Histological analysis

The mice were euthanized by standard decapitation 10 days after gene transfection. To evaluate the biosafety assessment of US treatment, vital organs (heart, liver, spleen, lung, kidney) were harvested and frozen. Take out the dehydrated tissue, drain the surface water with filter paper, smooth the target tissue with a scalpel, put the section face up on the specimen chuck, drop OCT embedding agent (Sakura, USA) around the tissue, put the specimen chuck on the quick-freezing table of the frozen section machine (CRYOSTAR NX50, Thermo, USA) for quick freezing and embedding. When OCT becomes white and hard, it can be sectioned. The specimen chuck was fixed on the slicer, the tissue surface was rough cut and then sliced. The slice thickness was 8–10 μM. the clean slide was placed on the top of the cut tissue slide, and the tissue was pasted on the slide. After the film is labeled, it should be stored at −20 °C for future use.

### Biochemical properties analysis

To investigate the changes in laboratory blood biochemistry indexes, whole blood and blood serums were gained from the eyeballs of mice from each group 48 h after treatment. The liver functions serum glutamic-pyruvic transaminase (ALT) and glutamic-oxalacetic transaminase (AST), as well as blood urea nitrogen (BUN) was tested at the clinical laboratory of the Third Affiliated Hospital of Guangzhou Medical University (Guangzhou, China).

### Statistical analysis

Statistical analyses were performed using GraphPad Prism 8 software (GraphPad Software Inc., La Jolla, California). Data were expressed as mean ± standard deviation. Each experiment was performed independently three times. Group comparisons were performed using Student’s *t*-test. *p*-Value < .05 was considered indicative of a statistically significant difference.

## Results and discussion

### Characterization and acoustic properties of miR-34a-mimic-FA-MBs

**Figure 1.**
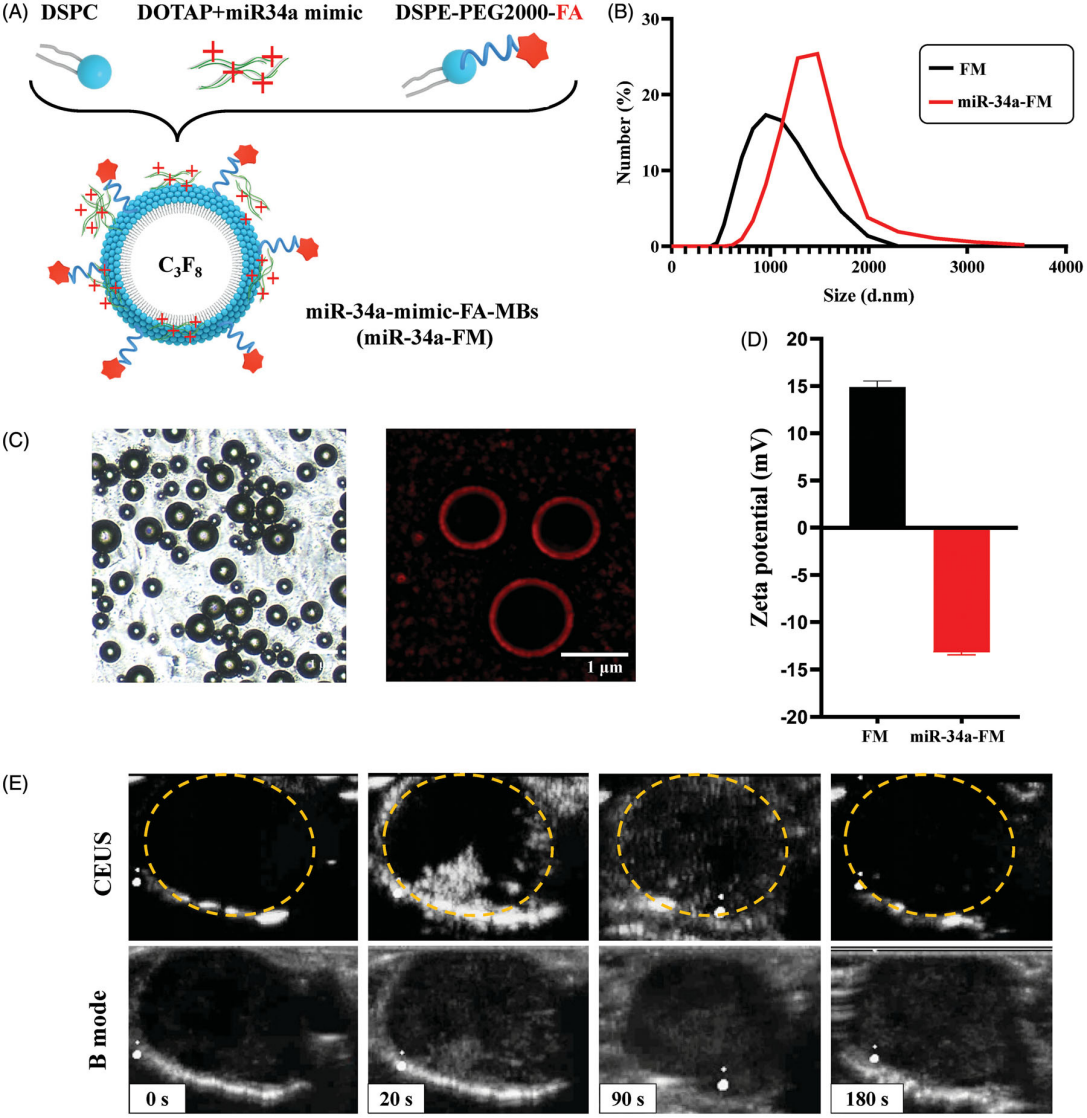
Preparation and characteristics of miR-34a-mimic loaded folate modified microbubbles (miR-34a-FM). (A) Schematic illustration of the fabrication of folate receptor modified microbubbles. (B) Size and concentration of miR-34a-FM. (C) miR-34a-FM adsorb with cytoplasmic membrane and Cy5-miR-34a-mimic loaded with MBs. (D) Zeta potential of miR-34a-FM. (E) Contrast image and B-mode image of miR-34a-FM in tumor-bearing mice.

As a miR-34a-mimic vehicle, miR-34a-FM maintains an MB structure (Figure 1(A)). The mean diameter of miR-34a-FM was 1.5 ± 0.3 μm (Figure 1(B)). MBs could attach well to cells and the spherical mimic (tagged by Cy5 dye) signals of MBs’ morphology suggested successful loading of miR-34a-mimic onto FM (Figure 1(C)). The dramatic decrease in the zeta-potential of FM from positive to negative after miR-34a-mimic loading (+14.9 ± 0.62 mV to −13.2 ± 0.3 mV) also confirmed the gene coating of the MBs’ shell (Figure 1(D)). To detect the US imaging capability of these targeted microbubbles, tumor-bearing mice were used for CEUS *in vivo*. The difference in the value of signal intensity represents the number of targeted bound MBs and the difference in signal intensity significantly increased after administration of miR-34a-FM, with fading of the signal strength starting after 180 s (Figure 1(E)).

### Optimization of US parameters for drug delivery and cell viability

**Figure 2.**
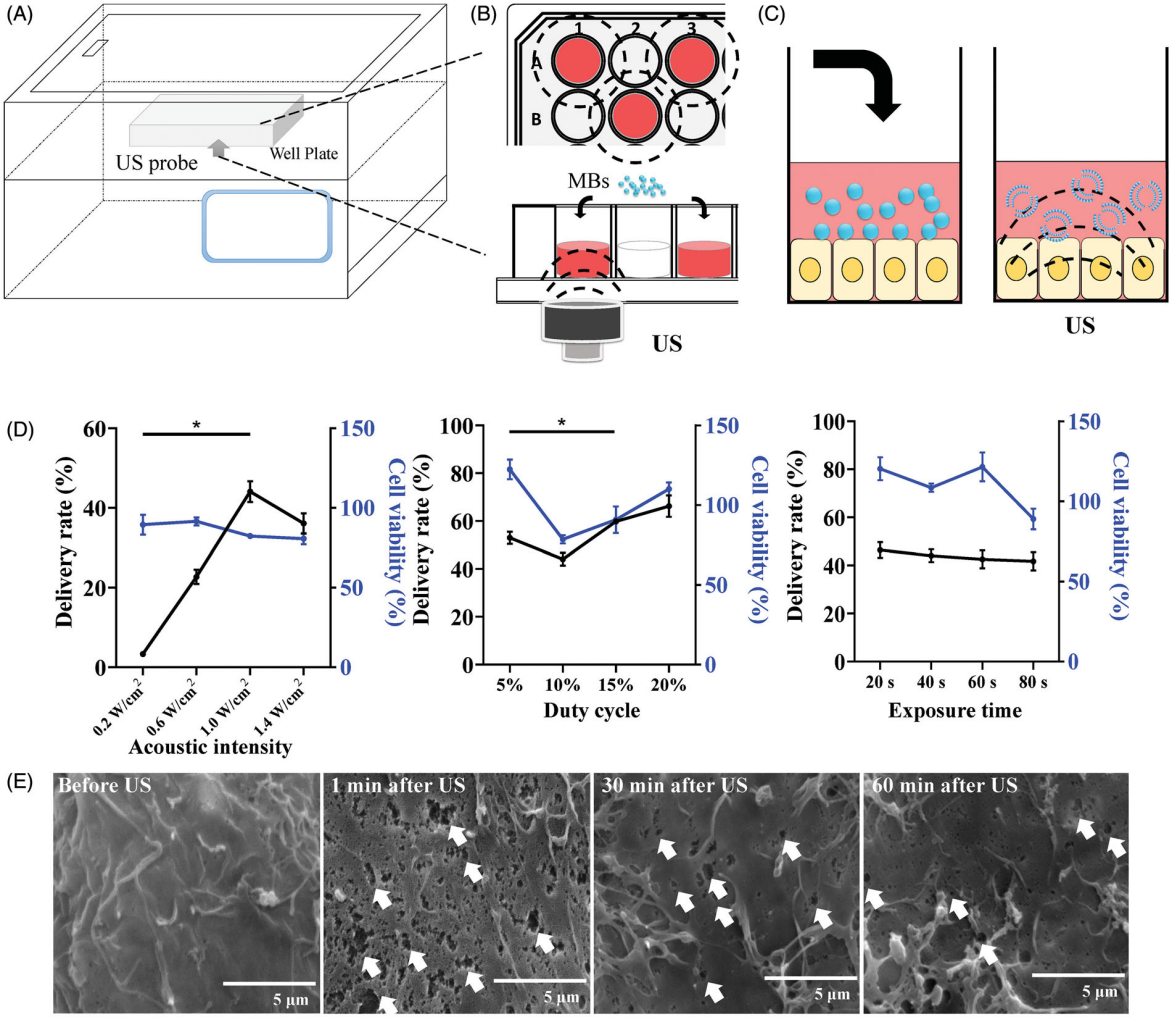
Illustration of the US system. (A) 24 well plate irradiated by US system. (B) cells were cultured onto the plate at every second row and column. (C) MBs collapsed after being exposed to US and induced cavitation. (D) Effect of FITC-dextran delivery and cell viability under different ultrasonic parameters including AI, DC, and ET. Data are presented as the mean ± standard deviation (*n* = 3). *p-Value < .05 compared with the control group. (E) The morphological changes of the cell membrane after the effect of cavitation.

The ultrasonic parameters (AI, ET, and DC) were optimized in our study. The 24-well plates were placed above the probe of the US system (Figure 2(A)) and SK-OV-3 cells were cultured onto the plate, every second row and column, in order to prevent interaction of US irradiation to each other (Figure 2(B)). The MBs collapsed after being exposed to the US and generation of cavitation cells was induced (Figure 2(C)). The effects of ultrasonic parameters on delivery efficiency and cell viability were investigated by flow cytometry and CCK-8 assay. The US device has a control panel to set different ultrasonic parameters (Figure S1(A)). The delivery rate of model molecules (FITC-dextran) was related to the opening degree of sono-pores of the cell membrane. When AI was greater than 1.0 W/cm^2^, with a fixed 10% DC, 20 s ET, the delivery rate of FITC-dextran decreased significantly, and AI had no obvious effect on cell viability. When AI was fixed at 1.0 W/cm^2^, and ET was fixed at 20 s, the point where two curves intersect reflects DC with 15% which was the most appropriate for delivery efficiency and cell viability. When AI was fixed at 1.0 W/cm^2^ and DC fixed at 10%, ET had no obvious effect on the delivery rate of FITC-dextran and cell viability. Because sonoporation was a transient physical process and miR-34a-FMs were observed to completely explode within 20 s, a minimum value of ET was set to 20 s (Figure S1(B)). Finally, we chose 20 s ET, 1.0 W/cm^2^ AI, and 15% DC as the optimal ultrasonic conditions in subsequent UTMD studies.

### The mechanism of UTMD-mediated drug delivery

Efficient gene introduction mediated by UTMD was dependent on the cavitation induced by the interaction between US and MBs. Studies have demonstrated that UTMD could enhance the permeability of the cell membrane and increase the delivery of genes to cells (Shapiro et al., 2016; Xie et al., 2016; Bez et al., 2019; Liufu et al., 2019). During cavitation, a strong shock wave and microstreaming were produced, which generated nanoscale pores on the cell surface (Rong et al., 2018; Li et al., 2021). SEM was performed to assess the alterations of membrane morphology in our UTMD experiments. As shown in Figure 2(E), compared with the untreated group (before the US), the group treated with the US immediately displayed numerous nanoscale pores on the cell membrane in response to UTMD. As time passed, the cell membrane sealed, and the number of sono-pores decreased. The cell membrane displayed close to complete recovery after 60 min and the morphology of cells returned to normal, as shown in our study.

### UTMD mediated miR-34a-mimic delivery to SKOV3 cells in vitro

To meet the urgent demand for UTMD studies, we developed a US transfection device (SXUltrasonic, Shenzhen, China), which is specifically designed for in vitro applications. The apparatus is easy to operate, allows for the fine-tuning of parameters (AI, ET, and DC), and cannot be easily contaminated because it is equipped with a sterile compartment. To our knowledge, most of the ultrasonic devices we have mentioned above employ handheld probes, which could directly increase the instability of the experiment result. In contrast, the inner structure of SXUltrasonic consists of a fixed probe underneath and a non-sound absorbing panel, ensuring the controllability of the experiment while reducing the interference of human factors.

**Figure 3.**
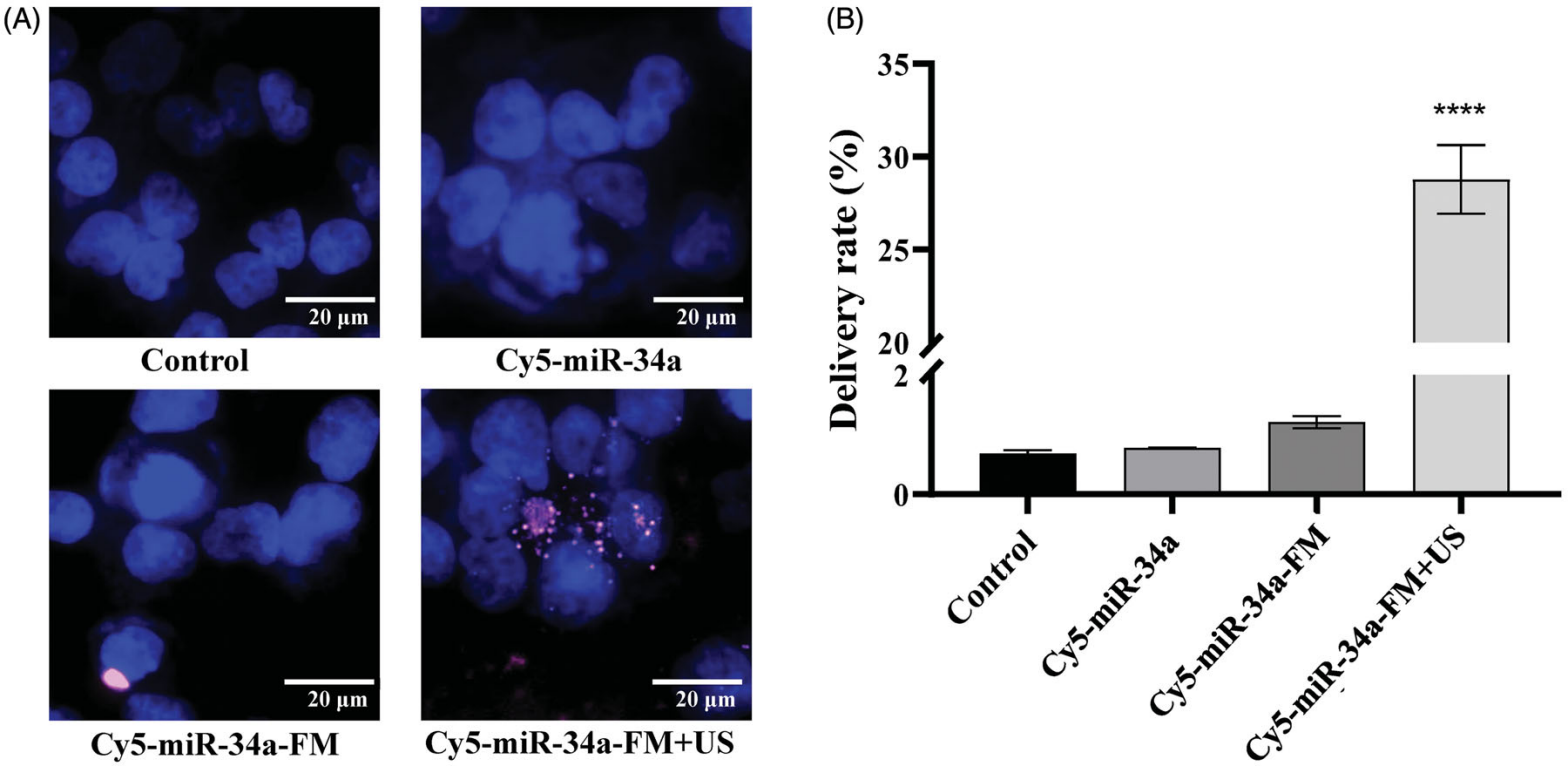
UTMD mediated miR-34a-mimic delivery *in vitro.* (A) Cellular uptake of a Cy5-labeled miR-34a-mimic (red) after different treatments. Cells left untreated, transfected with US + the miR-34a mimic and transfected with NBs + the miR-34a mimic were used as controls. Cells fixed on glass slides were stained with DAPI and visualized under a Panoramic MIDI scanner. Cell nuclei were stained blue by DAPI. Scale bars are 20 μm.

We have obtained the optimal result with 1.0 W/cm^2^ AI, 15% DC, and 20 s ET as the optimal ultrasonic conditions. Cy5-miR-34a-FM was synthesized and used in this experiment and after US treatment, the number of Cy5-positive cells increased significantly compared with other groups. The images in Figure 3(A) indicated that Cy5-labeled miR-34a-mimic (red) could enter cells with the aid of UTMD, rather than by Cy5-miR-34a alone or Cy5-miR-34a-FM without US group. The results from flow cytometry were also consistent with the results from fluorescence imaging. The transfection rate, which was represented by the percentage of Cy5-positive cells among the total cells, was greater than 28.4 ± 4.8% (Figure 3(B)). Therefore, UTMD-mediated miR-34a-mimic internalization was more efficient than passive diffusion and nonspecific targeting.

### MiR-34a induced apoptosis and invasion inhibition of SKOV3 cell in vitro

**Figure 4.**
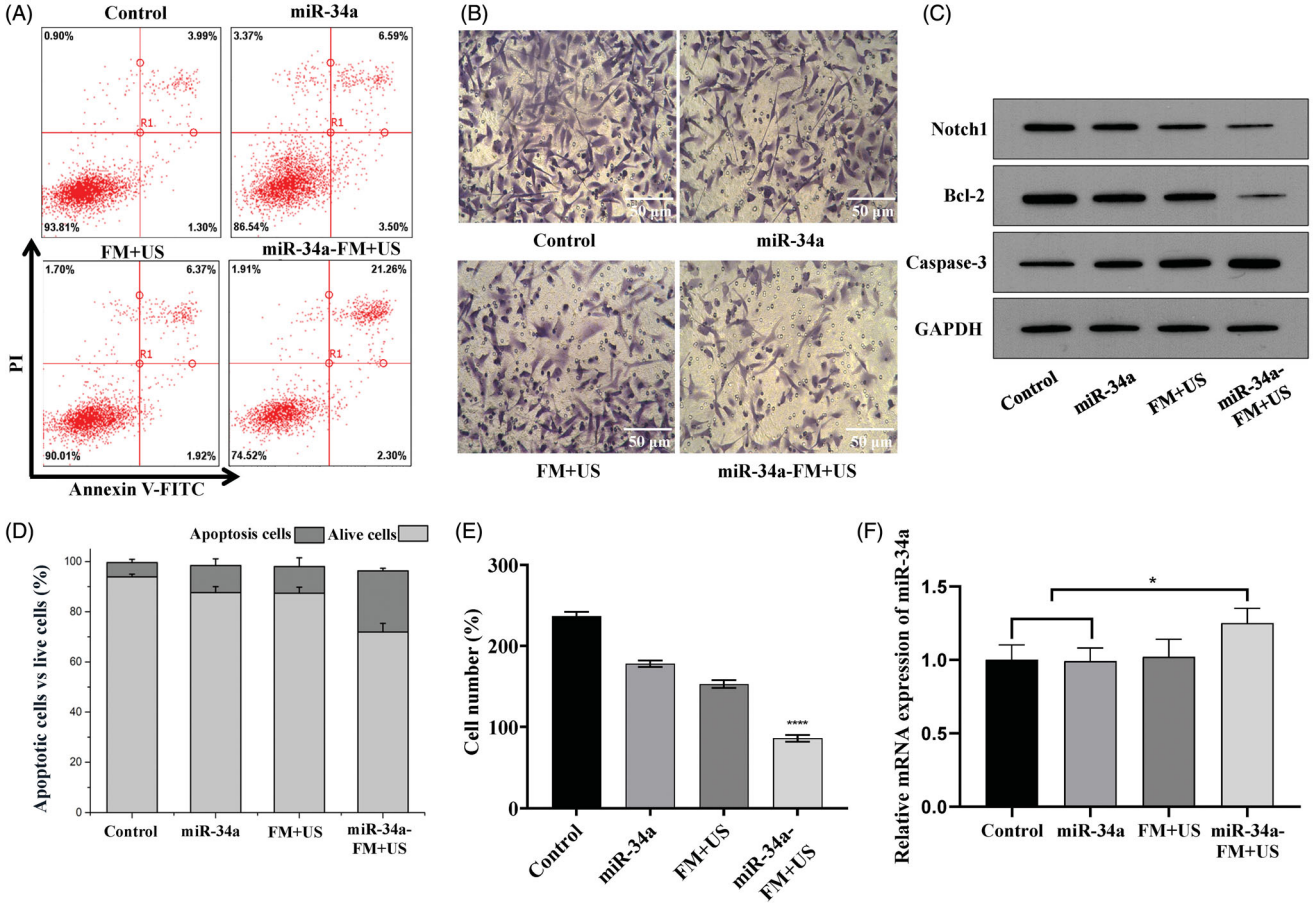
Apoptosis in SK-OV-3 cells induced by different treatments. (A) Apoptosis induction by miR-34a-mimic, FMB + US and miR-34a-FM + US was determined and compared by Annexin V/PI staining and (B) Cell invasion assay was used to analyze the invasiveness of cells. (C) Analysis of Notch1 expression in SK-OV-3 cells. Notch1 and Bcl-2 protein expression was analyzed by Western blot 48 h after microRNA transfection by UTMD. (D) Quantitative analysis by flow cytometry. (E) Quantitative analysis of cell number after cell invasion assay. (F) mRNA level after miR-34a-mimic delivery. *p-Value < .05, relative to the control group.

In our previous study, miR-34a-mimic was transfected by our UTMD method to inhibit the proliferation of breast cancer cells and induce apoptosis at the cellular level. The results showed that miR-34a-mimic delivered by UTMD efficiently inhibited cancer cell proliferation. In addition, downstream proteins including Notch1 and Bcl-2 were downregulated by miR-34a (Zhang et al., 2020). We evaluated the impact of UTMD-mediated miR-34a-mimic delivery for ovarian cancer cell apoptosis by Annexin-V/PI staining and flow cytometry. After 24 h of treatment, the total apoptosis rate was analyzed by flow cytometry. As shown in Figure 4(A,B), miR-34a-FM + US induced the highest rate of apoptosis, free miR-34a-mimic and FM + US produced similar rates of apoptosis. In addition, UTMD could induce apoptosis in some cells, which might account for the decreased cell viability of cancer cells and the results supported the idea that UTMD and gene transfection had a synergistic effect (Zhou et al., 2013).

Furthermore, intracellular expression levels of miR-34a in the miR-34a-FM + US group were upregulated, and incubation of cells with miR-34a-mimic could not upregulate the mRNA level of miR-34 (Figure 4(C)). The expression level of Notch1 was examined in this study and the result of Western blot analysis showed that the overexpression of miR-34 resulted in the suppression of Notch1 in SK-OV-3 cells. In recent years, it has been found that the abnormal expression of Notch1 signaling was closely related to the occurrence and development of several tumors (Jackstadt et al., 2019). Therefore, Notch1 has been demonstrating potential as a new approach to the prevention and treatment of tumors. Next, we sought to determine the effects of apoptosis triggered by Notch1 silencing in SK-OV-3 cells which was allied with the downregulation of Bcl-2 and upregulation of Caspase-3 (Figure 4(D)). The effects of miR-34 overexpression were also examined on the invasion of the SK-OV-3 cancer cells. It was found that miR-34 overexpression led to a significant (*p <*** **.01) suppression of SK-OV-3 cell invasion (Figure 4(E)). The invasion of miR-34a-FM + US-treated group was inhibited by 64% relative to the NC transfected cells (Figure 4(F)).

### UTMD mediated miR-34a-mimic delivery for ovarian cancer therapy in vivo

**Figure 5.**
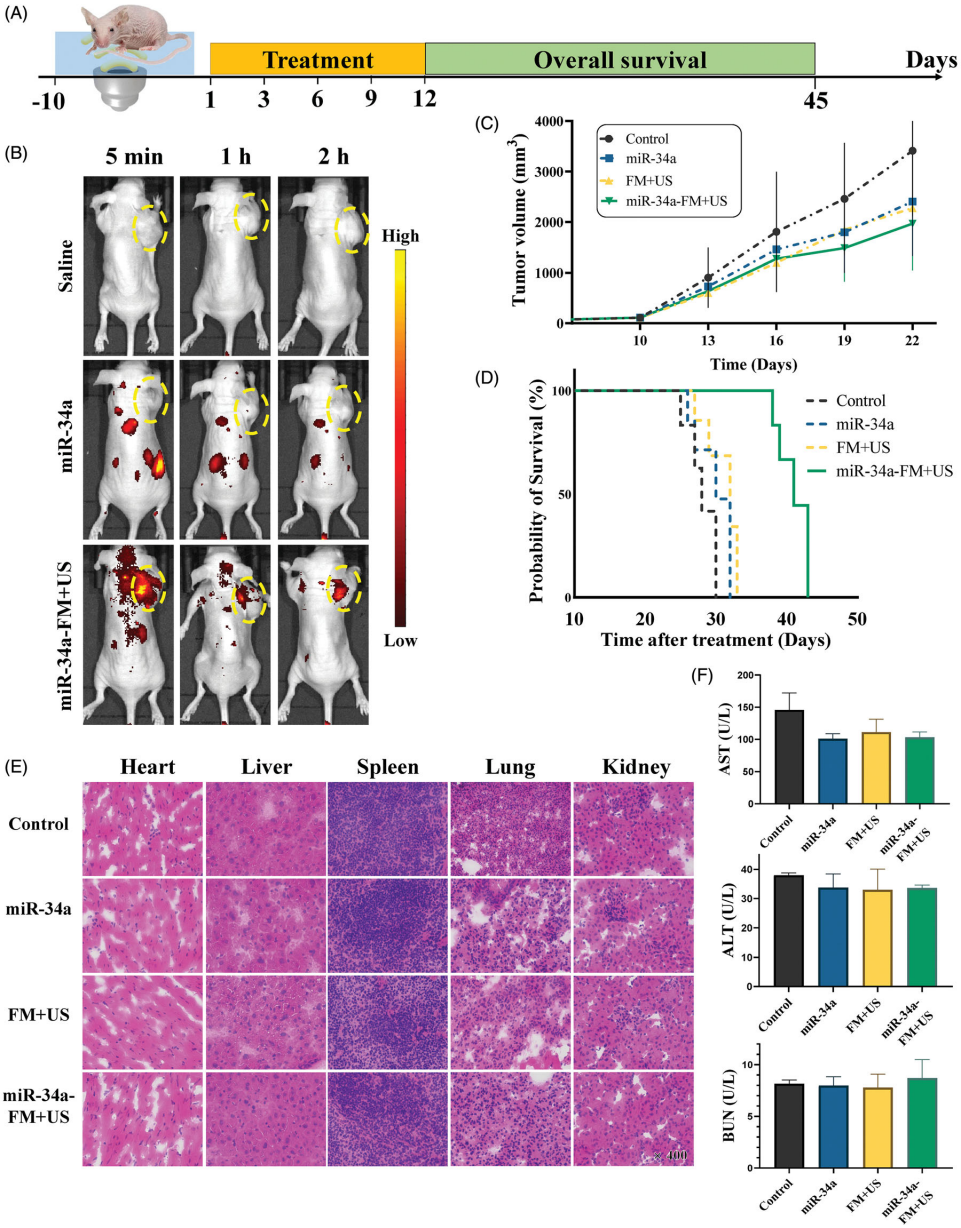
*In vivo* gene transfection. (A) The whole *in vivo* treatment trial process. (B) Tumor sections from these treated mice were observed under a fluorescence microscope. Scale bar: 50 µm. (C) The tumor growth curves within 22 d. *p-Value < .01. (D) Mouse survival curves from different groups treated with saline, miR-34a-mimic, FMB + US, or miR-34a-FM + US. (E) H&E staining of tumors from mice treated with saline, miR-34a-mimic, FMB + US, or miR-34a-FM + US. (E) The liver functions (ALT and AST) and the renal functions (BUN).

A subcutaneous tumor model was established to evaluate the antitumor effects of various formulations of groups *in vivo*. When the tumor volume reached 100 ∼ 120 mm^3^, to examine the *in vivo* miR-34a-mimic delivery mediated by UTMD, tumor-bearing mice received administration of saline, free Cy5-miR-34a-mimic, and Cy5-miR-34a-FM through tail-vein injection, followed by US irradiation. The treatment process of mice was shown in Figure 5(A). Every 3 days, the mice were weighed and the tumor volumes were measured. The *in vivo* fluorescence images after delivery revealed that tumors treated with Cy5-miR-34a-mimic and US had significantly higher fluorescence signals than those tumors which received free Cy5-miR-34a-mimic (Figure 5(B)). There was no fluorescence observed in the tumors treated with saline, with the presence of background fluorescence. The mice that received the free miR-34a-mimic showed background fluorescence at the tumor site, indicating that US and MBs were necessary to trigger sonoporation. Figure 5(C) showed the antitumor efficacy after UTMD-mediated miR-34a gene therapy. There were some antitumor effects in the mice that received free miR-34a-mimic and FM + US (UTMD) alone. As expected, an obvious tumor growth inhibition effect was observed in the miR-34a-FM + US group compared with the miR-34a-mimic and UTMD groups. No significant tumor growth inhibition was observed in the saline group. Meanwhile, the miR-34a-FM + US group had the longest survival time, reaching 43 days, while the other groups died after 30 days (Figure 5(D)).

### Histological analysis and biochemistry indexes

After all the treatments, the mice were sacrificed for the subsequent histopathological study of the tumors and vital organs to evaluate their efficacy and safety. The frozen sections from the heart, liver, spleen, lung, and kidney of these tumor-bearing mice, which supported the relatively normal histological structure, confirmed that there was no obvious organic damage or side effects, especially in the miR-34a-FM + US group (Figure 5(E)). Because of the deep location of the ovaries, US has traditionally been the most used tool for diagnostic purposes. Clinical studies have also shown that US demonstrated obvious efficacy in the treatment of tumor diseases due to its noninvasive ability to mediate precise drug delivery (Dimcevski et al., 2016). The tumor therapy results of this study indicated that miR-34a-FM combined with US irradiation possessed a good anti-tumor therapeutic effect on SKOV3 tumor-bearing mice. Hence, UTMD combined with miR-34a-mimic would be a promising non-invasive treatment, especially for lesions invading the adnexa uteri.

## Conclusion

We developed a safe and effective delivery strategy for miR-34a-mimic by US combined with folate receptor-modified MBs (miR-34a-FM). Taking advantage of UTMD to quickly trigger cell membrane perforation which mediated gene delivery, miR-34a-mimic could be used for the delivery and treatment of ovarian cancer. Optimization of parameters and improvement of the device improved the gene delivery effect. Overexpression of miR-34a in ovarian cancer cells confirmed that down-regulation of Notch1 expression activated the apoptotic pathway and inhibited cell migration. In subcutaneously transplanted mice, miR-34a-mimic was delivered to tumor tissue via UTMD, inhibiting tumor growth and prolonging the survival time of mice. This provides potential clinical applications for patients with advanced ovarian cancer with liver involvement.
